# Marked improvement in the success rate of medical management of early pregnancy failure following the implementation of a novel institutional protocol and treatment guidelines: a follow-up study

**DOI:** 10.1007/s00404-016-4179-6

**Published:** 2016-08-23

**Authors:** V. Colleselli, T. Nell, T. Bartosik, C. Brunner, A. Ciresa-Koenig, L. Wildt, C. Marth, B. Seeber

**Affiliations:** 1 Department of Gynecology and Obstetrics, Medical University of Innsbruck, Anichstrasse 35, 6020 Innsbruck, Austria; 2 Department of Gynecologic Endocrinology and Reproductive Medicine, Medical University of Innsbruck, Anichstrasse 35, 6020 Innsbruck, Austria

**Keywords:** Early pregnancy failure, Medical management, Mifepristone, Misoprostol

## Abstract

**Purpose:**

To analyze the success rate, time to passage of tissue and subjective patient experience of a newly implemented protocol for medical management of early pregnancy failure (EPF) over a 2-year period.

**Methods:**

A retrospective chart review of all patients with early pregnancy failure primarily opting for medical management was performed. 200 mg mifepristone were administered orally, followed by a single vaginal dose of 800 mcg misoprostol after 36–48 h. We followed-up with our patients using a written questionnaire.

**Results:**

167 women were included in the present study. We observed an overall success rate of 92 %, defined as no need for surgical management after medication administration. We could not identify predictive values for success in a multivariate regression analysis. Most patients (84 %) passed tissue within 6 h after misoprostol administration. The protocol was well tolerated with a low incidence of side effects. Pain was managed well with sufficient analgesics. Responders to the questionnaire felt adequately informed prior to treatment and rated their overall experience as positive.

**Conclusion:**

The adaption of the institutional medical protocol resulted in a marked improvement of success rate when compared to the previously used protocol (92 vs. 61 %). We credit this increase to the adjusted medication schema as well as to targeted physician education on the expected course and interpretation of outcome measures. Our results underscore that the medical management of EPF is a safe and effective alternative to surgical evacuation in the clinical setting.

## Introduction

Early pregnancy failure (EPF) is a common pregnancy complication with approximately 25 % of pregnancies ending in miscarriage and with 1 in 4 women experiencing this problem during her reproductive lifespan [1–3]. With the routine use of ultrasound, these pregnancy failures are often diagnosed prior to the onset of any symptoms, such as bleeding or cramping, and have been therefore termed “missed abortions”. Improvements in ultrasound technology have further enabled the subclassification of asymptomatic EPF into intrauterine embryonic/fetal demise (IUED/IUFD) and anembryonic gestation (“blighted ovum”). In contrast, women with inevitable abortion, incomplete abortion and completed abortion experience cervical dilation, cramping, and bleeding during the passage of tissue that ends in miscarriage [4, 5].

Considering the psychological and physical burden of experiencing a pregnancy failure, it is important to be able to offer patients effective, timely, and safe management [6, 7]. Upon the definitive diagnosis of EPF, the following three treatment options may be considered: (1) expectant management with follow-up; (2) surgical management with pregnancy evacuation [cervical dilation followed by suction or blunt curettage (D&C)]; or (3) medical management using misoprostol or a combination of mifepristone and misoprostol to induce uterine evacuation [4, 5].

Historically, surgical management was the mainstay of management, offering prompt uterine evacuation with a high rate of success of over 95 %. More recently, medical management has become an established alternative option for patients wishing to avoid surgery and its associated operative risks, such as uterine perforation, endometritis, injury to the cervix, and Asherman’s syndrome, as well as potential anesthesia-related complications [4, 5, 8–11]. Medical management of EPF is routinely carried out with the prostaglandin E1 analogue Misoprostol which induces cervical dilation and uterine contractions, inducing the vaginal expulsion of the failed pregnancy. Some, but not all, clinicians administer mifepristone, a competitive progesterone antagonist, 24–48 h prior to misoprostol administration to improve the success rate by disrupting the progesterone-mediated trophoblast-decidua interaction. Corresponding study results are contradictory. While some studies show promising results exceeding a success rate of 85–90 % with coexistent mifepristone use, others have found no additional benefit whether a dose of 200 mg or 600 mg is given, at the cost of increased expense [10, 12–17]. Unlike its beneficial use in elective terminations of viable pregnancies (elective abortions), mifepristone may have limited usefulness in failed pregnancy which have lower progesterone levels [18–21].

The overall success rate of medical management quoted in the literature is highly variable, ranging from 66–83 % in clinical practice and even as high as 95 % in small research studies, as summarized in Table 1. In our previous publication of the success rate in routine clinical practice in our university clinic, we found a disappointingly low rate of only 61 % [14]. The reason for this discrepancy is likely due to the great institutional variability in the medications, their dosages, routes, and time intervals of administration used for treatment. In addition, as shown in Table 1, the definition of successful treatment varies greatly from study to study, and has been defined as no presence of gestational sac on ultrasound, termination of vaginal bleeding, or by endometrial thickness on ultrasound [5, 22–25]. Finally, the more experienced clinician is in the use of medical management, the more comfortable he/she may be with expectantly managing the patient with heavy or prolonged bleeding post-medication administration. Less experienced clinicians may be prompted to intervene too quickly with curettage, thus decreasing the perceived success rate.

**Table 1:** Protocols in the literature

Study	Design	Mifepristone	Misoprostol	Success	Definition of success
Colleselli et al. [14]	Retrosp. *N* = 168	600 mg orally	400 mcg orally, followed by 400 mcg vaginally in 4 h intervals, max. 2400 mcg	61 %	Not standardized, Dependent on treating physician
Van den Berg et al [10]	Retrosp. *N* = 301	Group 1: 200 mg orally	Group 1: after 36 h 800 µg vaginally Group 2: 2 doses of 800 µg vaginally, time interval 24 h In both groups additional 800 µg vaginally if no bleeding or cramping after 24 h	Group 1: 67 % Group 2: 55 % (statistically significant)	Clinical signs, empty uterine cavity on ultrasound or hysteroscopy, absence of products of conception in histology
Barcelo et al. [27]	Retrosp. *N* = 946	–	2 doses of 600 µg or 800 µg vaginally, time interval 24 h	88/91 %	no gestational sac on ultrasound
Kollitz et al. [13]	Retrosp. *N* = 123	200 mg orally	After 24 h 800 µg vaginally, if indicated additional dose after 7 days	80/83 %	No presence of gestational sac and endometrial thickness <30 mm on ultrasound
Stockheim et al. [16]	Prosp. *N* = 115	Group 1: 600 mg orally	Group 1: after 48 h 800 µg orally Group 2: 2 doses of 800 µg orally, time interval 48 h	Group 1: 66 % Group 2: 74 %	No need for surgical intervention
Schreiber et al. [12]	Prosp. *N* = 30	200 mg orally	After 24 h 800 µg vaginally, if indicated additional dose after 7 days	90/93 %	Expulsion of gestational sac, no need for D&C
Zhang et al. [30]	Prosp. *N* = 652	–	800 µg vaginally, if indicated additional dose after 48 h	71/84 %	no need for surgical intervention within 30 days after initial treatment
Grønlund et al [15]	Prosp. *N* = 176	Group 1: 600 mg orally	400 µg vaginally, if no bleeding after 2 h à 200 µg additionally (group 1: 48 h after mifepristone)	Group 1: 4 % Group 2: 71 %	No need for surgical evacuation after medical treatment

*Retrosp.* retrospective, *prosp.* prospective

**Fig. 1:**
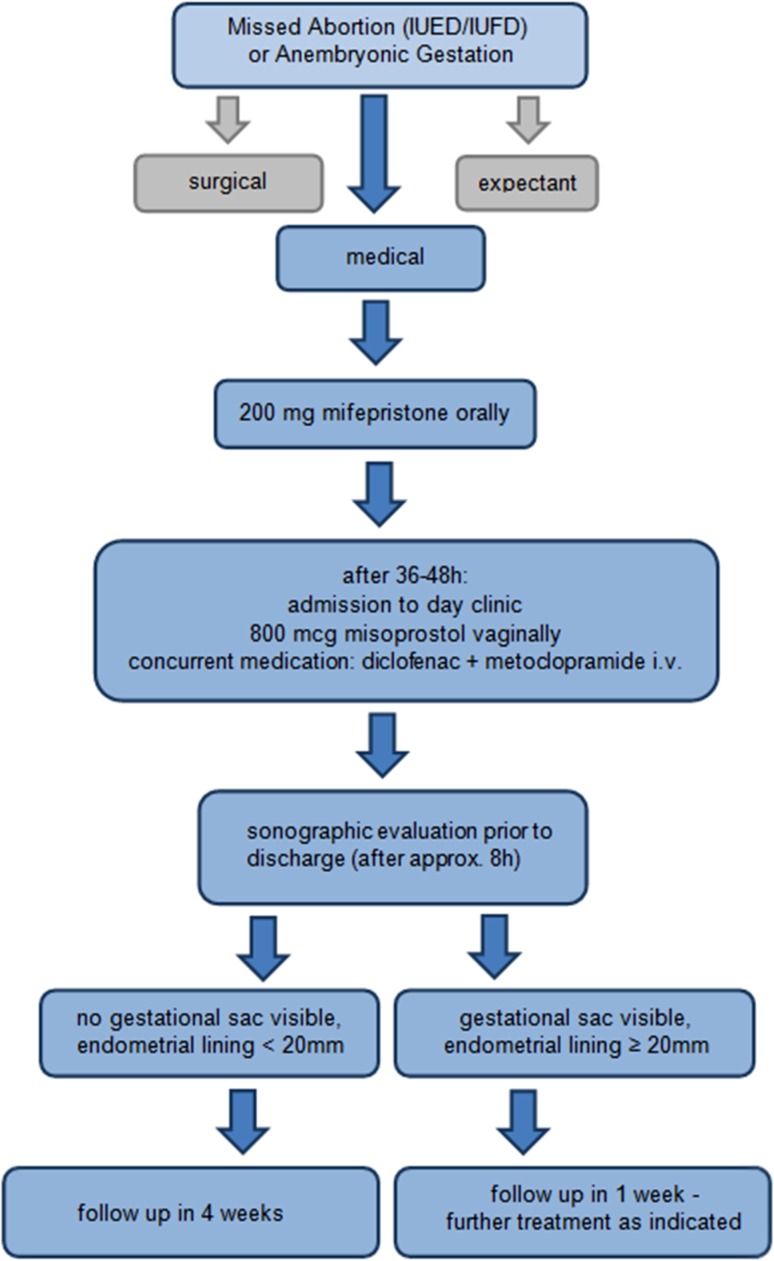
Implemented protocol

We aimed to improve the success of medical management of EPF in our university hospital setting by adopting the most successful evidenced-based medication regimen while limiting costs. In addition, we formally reviewed with the treating physicians, the expected effects of treatment (bleeding pattern and duration, cramping, and pain) as well as the expected post-treatment ultrasound findings and their correct interpretation. Finally, we defined standard operating procedures (SOP) to standardize and optimize the clinical course of treatment and to define the indications for surgical intervention. This treatment algorithm is shown in Fig. 1.

In this study, we retrospectively analyze the success rate of the medical management of EPF in the first 2 years upon the implementation of the above-mentioned changes. In addition, we report the results of a questionnaire sent to the patients who were treated with medical management during this time period which compared the patients’ expectations of treatment (bleeding, pain, and side effects) to their actual experiences and inquired about their willingness to choose this treatment option in the future or to recommend it to a friend.

## Materials and methods

The present retrospective study was approved by the institutional ethics committee of the Medical University of Innsbruck. The participants who returned the follow-up questionnaires signed an informed consent.

As summarized in Fig. 1, according to institutional SOPs, all patients diagnosed with a non-viable early pregnancy are presented three options upon diagnosis: (1) expectant management, (2) surgical management by dilation and curettage (D&C), and (3) medical management with mifepristone and misoprostol. All patients primarily opting for medical management receive a single oral dose of 200 mg Mifepristone on an out-patient basis and are subsequently admitted 36–48 h later to our day-inpatient unit. On admission, an ultrasound is performed to confirm the continued presence of the intrauterine EPF, and diclofenac and metogastrone are given intravenously for prevention of pain and nausea, respectively. Subsequently, a single dose of 800 mcg (4 Tablets of 200 mcg each) misoprostol is administered intra-vaginally to the posterior fornix by the treating physician. All patients are monitored for the following 8 h for bleeding and passage of tissue which is recorded in the electronic medical record. They may be administered additional analgesics or antiemetics, as necessary. In a small subset of patients, outside of the defined SOP, an additional dose of 400 mcg Misoprostol was administered buccally when no passage of tissue took place after 6 h of the vaginal dose. Following the suspected passage of the failed pregnancy, or at the latest at 5 pm (closing time of the day-unit), a transvaginal ultrasound is performed to guide further follow-up. If the ultrasound shows no gestational sac present, the endometrial thickness is less than 20 mm, and bleeding is within normal limits, then the patient is discharged and an out-patient follow-up visit is scheduled in 4 weeks’ time. If the gestational sac is still visible and/or the endometrium thickness measures more than 20 mm, and bleeding is within normal limits, then the patient is discharged and scheduled for follow-up in 1 week. At the 1 week appointment, a re-evaluation is performed. If there are sonographic or clinical evidences for ongoing EPF, then the patient can opt for another course of misoprostol, expectant management, or surgical management.

Using admission records, we identified all patients who received medical management for EPF in the 2-year period between March 1, 2013 and February 28, 2015. To be included, the women had to have a missed abortion ≤12-week gestation (intrauterine embryonic or fetal demise or anembryonic gestation without cervical dilation or heavy bleeding). Exclusion criteria included multiple gestation, pregnancy with an IUD in place, gestational age >13 weeks by ultrasound, and the diagnosis of inevitable, incomplete, and complete miscarriage. Patients’ charts and electronic records were retrospectively abstracted to collect clinical and ultrasound data at initial presentation and the clinical course following misoprostol administration, focusing on the time to passage of tissue, amount of bleeding, and medications’ administered and documented side effects. Relevant previous obstetric, gynecologic, and medical history were recorded. Treatment success was defined as no surgical intervention after initiation of medical treatment. We calculated the overall success rate for all women, and according to gestational age (≤9 vs. ≥10), and diagnosis (anembryonic gestation vs. IUED/IUFD). Logistic regression analyses were performed to evaluate for pre-selected predictive factors of success, using the independent variables age, body mass index (BMI), parity, gestational age (GA), and diagnosis.

We compared the outcomes of the present study to those we previously reported from a retrospective review of cases between 2006 and February 2012 prior to the establishment of the current SOPs. We used the student’s *t* test or Mann–Whitney *U* test to compare parametric and non-parametric outcomes, respectively, and the Chi-square test to compare binomial outcomes. Analyses were performed using PASW Statistics for Windows, Version 18.0. Chicago: SPSS Inc.

Finally, we followed-up with our patients by mailing each of the women a questionnaire to be filled out and returned in an envelope provided. The questions in this questionnaire asked whether the treatment experience met the women’s expectations in terms of amount and duration of bleeding, amount of pain as well as asked how they rated the overall experience and whether they would choose this treatment method again and/or recommend it to a friend.

## Results

We identified 167 patients who met the inclusion criteria and were included in this study. Patient characteristics are shown in Table 2 and are similar to those of the women who made up the study population reported in our previous publication [14].

**Table 2:** Patient characteristics

Parameter	March 2013–February 2015 *N* = 167		Colleselli et al. [14] *N* = 168		*p*
	Mean ± SD	Median (min.–max.)	Mean ± SD	Median (min.–max.)	
Age (years)	33.2 ± 6.0	34 (18–47)	32.7 ± 6.6	33 (16–45)	0.474
BMI	23.6 ± 4.2	23.0 (15.5–37.8)	22.7 ± 3.7	22 (12.8–37.0)	0.085
Gravidity	–	2 (1–9)	–	2 (1–7)	–
Parity	–	1 (0–5)	–	0 (0–5)	–
GA by LMP (weeks)	10.0 ± 1.6	10 (6–15)	10.1 ± 2.1	10 (5–18)	0.756
GA by ultrasound (weeks)	7.8 ± 1.5	7 (5–12)	8.1 ± 1.8	8 (5–13)	0.343
Time to surgery after misoprostol (days)	26.1 ± 19.3	22.5 (0–57)	5.3 ± 9.6	1 (0–42)	<0.001*
Expulsion time after administration of misoprostol (hours)	4.7 ± 1.8	4.3 (1.5–12.5)	8.4 ± 7.2	5.5 (1–34)	<0.001*

* Statistically significant

153 of 167 women were successfully treated with medical management for a cumulative success rate of 92 % (*n* = 69/79 or 81 % in the first year and *n* = 84/88 or 97 % in the second year). Subgroup analyses by gestational age (≤9 weeks, ≥10 weeks) and diagnosis (IUED/IUFD vs. anembryonic gestation) showed no significant difference in successful management (GA by LMP—91 vs. 88 %, *p* = 0.498; GA by ultrasound—89 vs. 82 %, *p* = 0.468; diagnosis—87 vs. 97 %, *p* = 0.081).

To identify possible predictive factors for success, we performed a multivariate regression analysis. None of the pre-selected independent variables were able to predict successful treatment (Table 3).

**Table 3:** Multivariate regression analysis, independent variable = success

Parameter	*p*
Diagnosis^a^	0.253
Age^b^	0.449
BMI^c^	0.860
Parity^d^	0.811
GA by LMP^e^	0.564

^a^Diagnosis: IUED/IUFD vs. anembryonic gestation

^b^Age (years)-groups: <24, 25–64, 35–39, >40

^c^BMI groups: <18, 18–25, >25

^d^Parity: 0, ≥1

^e^GA by LMP (weeks): ≤9, ≥10

Since this is a retrospective evaluation of routine clinical practice, a small number of women were treated outside of the standardized protocol by the treating physician. Namely, in the absence of passage of tissue within 6 h of vaginal misoprostol administration, 26 patients (16 %) received an additional dose of 400 mcg misoprostol bucally. Of these women, 15 passed tissue within the following 2–3 h, while 11 still had no passage of tissue.

In 92 cases, the exact time of the first passage of tissue after administration of misoprostol was documented. In 77 of these women (84 %), it occurred within 6 h after misoprostol administration, with a median of 4.3 h. In the remaining 15 women, it was still within 9 h of medication administration, since these women were discharged without a gestational sac. Overall, the time to passage of tissue was statistically significantly shorter than with our previous protocol when the median time was 5.5 h. In contrast, the time to surgical intervention was statistically significantly longer with the revised protocol. In the few women who needed surgical evacuation, this was performed on average 26 days after medication administration, in comparison with just 5 days post-treatment between 2006 and 2012.

**Fig. 2:**
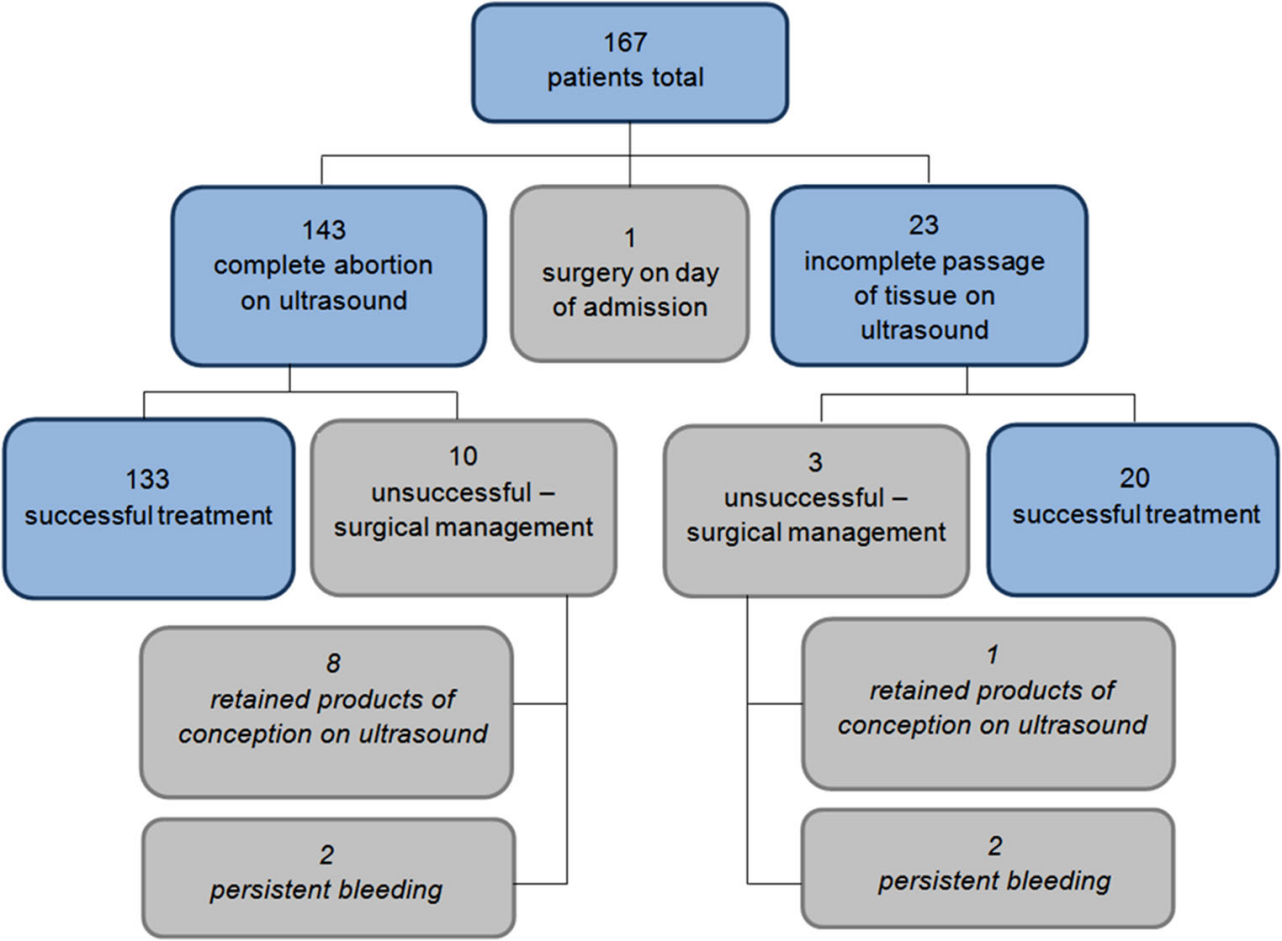
Outcome

In Fig. 2, we show the detailed outcomes for the patients. 23 patients were discharged with a visible gestational sac or endometrial lining >20 mm on ultrasound. Only 3 of these women were required a surgical intervention (curettage) to be subsequently performed. In 2 of these women this was due to persistent bleeding and in 1 of the women due to continued presence of the gestational sac on ultrasound. In none of these women were the surgical intervention emergent.

Of the 143 women discharged without a gestational sac and Endometrium <20 mm (presumed to be a completed abortion), ten nonetheless underwent surgical intervention. The indications for curettage in these women were suspected retained products of conception on ultrasound in eight cases and persistent bleeding in two cases. None of these interventions were performed on an emergency basis. None of these women opted for a repeat treatment with misoprostol. For the total of 13 women who needed curettage, the procedure was performed between 0 and 57 days after the administration of misoprostol, median 22.5 days. In 50 % of women, chorionic villi were obtained, confirming retained products of conception. One woman required a blood transfusion post-surgery for heavy intraoperative bleeding. No blood transfusion was administered after medical management alone.

The overall rate of side effects was low. The most common side effects reported were nausea (10 %), vomiting (5 %), diarrhea (4 %), cramping, and pain (59 %); however, most patients only experienced mild pain (90 %).

To gain insight into the subjective experience of our patients, we sent all medically treated patients a questionnaire together with a pre-paid return-addressed envelope, 62 of which were returned (return rate 37 %). 92 % of questionnaire participants felt that they received sufficient written and oral information prior to treatment. Nonetheless, fewer than half of the women reported the duration and amount of bleeding and pain to be consistent with expectations. In 27–34 % of responders, these parameters were more severe than expected, in the remaining responders less than expected, as shown in Table 4. Despite these results, 65 % of responders evaluated the overall experience as positive, with only 15 % negative and 21 % neutral ratings. 63 % of responders would opt for medical therapy again in the case of another EPF, while only 10 % would primarily choose surgery, and the remainder were not sure. Positive aspects of medical management most frequently noted were avoidance of a surgical procedure (76 %), care and support of staff (66 %), short treatment duration (57 %), and well-managed pain control (44 %). We saw an improvement in responses when comparing the first year to the second year of protocol implementation, as shown in Table 5. Due to only five questionnaires being returned from women with unsuccessful treatment, we were unable to make meaningful comparisons of their treatment evaluations compared to those treated successfully.

**Table 4:** Respondents’ expectations

	Consistent with expectations (%)	More than expected (%)	Less than expected (%)
Duration of bleeding	44	27	27
Amount of bleeding	48	34	18
Pain	40	31	29

**Table 5:** Questionnaire—comparison of year one and two

	March 2013–February 2014 *N* = 32 (%)	March 2014–February 2015 *N* = 30 (%)
Sufficient information	84	100
Duration of bleeding consistent with expectations	38	50
Amount of bleeding consistent with expectations	44	53
Pain consistent with expectations	34	47
Choose medical treatment again	60	67
Recommend medical treatment to a friend	69	87

## Discussion

The introduction of a new institutional protocol for the medical management of EPF resulted in a marked improvement in the success rate to 92 %, compared to the previous success of 61 % [14]. This high success rate is comparable to that reported in interventional studies under research protocols summarized in Table 1. For this improvement, we credit the use of an evidence-based medication schema (200 mg mifepristone orally followed by a single dose of 800 mcg misoprostol vaginally), targeted physician education, as well as the adoption of SOPs. The aim of the SOPs was to standardize the treatment algorithm and to aid the clinician in interpreting clinical signs and ultrasound results to plan follow-up accordingly.

Most women expelled the failed pregnancy during their stay in our day clinic and within only a few hours after medication administration. These data are important in accurately counseling women regarding their expectations. Although medical management can be safely performed as an out-patient treatment at home, we feel it is reassuring for the women to be monitored for bleeding and to be offered adequate pain management [26, 27].

Of the 23 women who did not expel the tissue during the first day of monitoring, the great majority (87 %) did so within the following week. This is consistent with previous studies and supports the recommendation to allow up to 7 days before subsequent re-evaluation and additional treatment [11]. This is a safe management strategy, supported by our findings that none of the women required an emergent surgical intervention due to heavy bleeding.

The most frequent indication for surgical intervention was persistent vaginal bleeding despite previous passage of the gestational sac. Very few women had sonographic evidence of persistent products of conception as the indication for surgery. Histological evaluation confirmed the presence if intrauterine chorionic villi in only half of the curettage specimens. Thus, it is impossible to know whether some or most of these women would have spontaneously ceased bleeding without surgical intervention. Previous studies have shown that bleeding patterns following medical management of EPF can be quite variable, but prolonged bleeding exceeding 20 days is not uncommon [28]. In fact, recent management strategies call for a longer time until intervention, waiting for up to 4–6 weeks [29].

It is reassuring that this combined protocol of mifepristone and misoprostol was well tolerated with a paucity of gastrointestinal side effects, especially when compared to other studies [30]. This may be partly due to the prophylactic administration of antiemetics prior to misoprostol administration.

Nonetheless, facing a non-viable pregnancy is psychologically difficult for affected women. Eligible women should, therefore, be counseled appropriately to enhance their understanding and expectations of treatment and psychological counseling should be offered routinely.

The study is limited by its retrospective design and lack of randomization. Therefore, we cannot evaluate whether the women who chose first-line medical management differ from those who prefer surgical treatment from the outset or from those who choose expectant management. However, due to strong patient preferences for which treatment they choose, a randomized study of medical vs. surgical intervention will likely never be able to be conducted. The study was conducted at a single university-based hospital, which may potentially limit the generalizability of the results to other treatment settings.

To further improve counseling for medical treatment, we designed a questionnaire to investigate patients’ experiences and satisfaction with the present protocol. Although a detailed written informed consent and information sheet is provided prior to treatment begin, one which the responders deemed adequately informative, their expectations regarding bleeding and pain were nonetheless incorrect in more than half of cases. This highlights the importance of an individual discussion with each patient. We did see a distinct improvement in the accuracy of expectations when comparing after the first year to the second year following the implantation of the protocol. We believe that this improvement might be attributed to more clinical experience and increasing familiarity with the SOPs, translating into more accurate information given to the patient.

Surgical treatment of EPF is associated with the risk factors of perforation, bleeding, anesthesia, and subsequent Asherman’s syndrome [8]. In addition, preliminary evidence shows that surgical management, especially with dilation of the cervix and curettage, might have adverse effects on future pregnancies [31–33]. Therefore, it is especially important to be able to offer highly effective medical management as an alternative to operative intervention. Medical management needs to be performed using evidence-based protocols by physicians trained in the expected outcomes, especially regarding bleeding and pain, and in the interpretation of ultrasound findings so as not to intervene unnecessarily.

In a standard gamble study, Griaziosi et al. showed that when faced with the diagnosis of EPF, women prefer medical management to surgical when the success rate of the former exceeds 65 % [7]. We were able to far exceed this success rate in our routine clinical practice and without adherence to a strict research protocol. In conclusion, our results support the use of medical management as a valuable non-invasive alternative to surgery in routine clinical practice. Our findings, furthermore, underscore the importance of formally educating caregivers regarding the expected findings, expected pain, patterns of bleeding following administration of medications and ultrasound interpretation to optimize treatment success.
